# Book Reviews

**Published:** 1817-05

**Authors:** 


### CRITICAL ANALYSIS

### OF RECENT PUBLICATIONS,

IN THE

### DIFFERENT BRANCHES OF PHYSIC, SURGERY, AND MEDICAL PHILOSOPHY.

*Additional Reports on the Effects of a Peculiar Regimen in Cases of Cancer, Scrofula, Consumption, Asthma, and other Chronic Diseases.* By WILLIAM LAMBE, M.D. Fellow of the Royal College of Physicians. 8vo.—Pp. 526. Mawman, London, 1815.

OUR readers will recollect this candid and eloquent writer. The present may be truly said to be a counter-part of his former labours, written with the same earnestness, the same benevolent intentions, the same display of learning and modern reading, and we feel obliged to add, the same adherence to a system. In the preface, we are informed, that he has purposely avoided all refined reasonings about the nature of the matter which, insinuating itself into the body in unsuspected vehicles, undermines its powers and lays the foundation of fatal diseases.

"It is not (says Dr. L.) that I think any thing which I have formerly advanced on these subjects untenable or visionary. In fact, the more I have considered the subject, the more have I been convinced of the general correctness of the opinions I have delivered. But several experiments which I have made, are still unfinished; other employment, particularly the attention due to this publication, having occupied my time. When I have completed the inquiries, in which I have engaged, I shall probably publish them in a separate form. This may be more useful, than blending matters more strictly scientific with things designed for the general reader and common utility."

Whilst we cannot help wishing, that the present publication had been delayed till these facts were ascertained, we are forced to admire the good intentions of the writer, who, from a sense of duty, ventures to offer his doctrines to the world without all the support he conceives they will ultimately receive.

The first chapter begins with some notice of the manner in which his former work was received; by some, with candor to the author and advantage to themselves; by others, in a manner which it is evident the author feels more than he ought, or than he is willing to believe. Some very fair remarks follow on the imperfect state of medicine, practical and theoretical, on the similarity of disease in all ages, and on the erroneous reasoning of physicians, founded, for the most part, on the erroneous philosophy of their day. This concludes with some compliments to Hippocrates, which, how much soever they may be deserved, we conceive are, in this instance, misplaced. We have always considered that difference which Hippocrates remarked in the inhabitants of different countries, and the similarity of those in the same district, rather as the effect of consanguinity preserved from the patriarchal manners, than from either the air, the water, or any other locality; and we are confirmed in this opinion, by reflecting that all these similarities lessen in proportion as a race becomes more mixed.

The preceding chapter commences with what is always considered an unfair mode of reasoning, viz. an inquiry why man is made obnoxious to so many physical evils.

"It has been taught, both by antient and modern philosophers, that the universe is, upon the whole, a perfect work; or the best that could have been possibly made. It has been hard, however, to reconcile the existence of evil with this hypothesis; and those, who have attempted to solve this knotty problem, have contented themselves with supposing, that it has been the result of some inevitable necessity. One of the antient sages adopted this explanation to account for the diseases of men. Crysippus was of opinion, that it could never have been the aim or first intention of the Author of nature, and Parent of all good, to make men obnoxious to diseases; but that while he was producing many excellent things, and forming his work in the best manner, other things also arose, connected with them, that were incommodious: which were not made for their own sakes, but were permitted, as necessary consequences of what was best."

However unsatisfactory all reasoning must be on subjects which we never can comprehend, we are better satisfied with it than with Dr. Lambe's remarks.

"This certainly (says he,) does not appear to be entertaining very exalted notions of divine power. To suppose, either that diseases are not real evils, or to feign an hypothetical necessity for their existence, and to pronounce it impossible for omnipotence itself to preserve the human body from them, (for this account involves, I think, one of these suppositions,) appears an equal extravagance.

"When we consider the tendency of nature to perfection in all her works, and that this tendency is in nothing more apparent, than in the structure of animal bodies, it appears indeed a strange anomaly, that the human frame, the master-piece of the creation, should be so liable to derangement and disease. If I may say so without irreverence, it appears as if the most beautiful of designs had failed from error and want of wisdom in the execution. More than than half the race perish in infancy, and, of the remainder, a large portion are the victims of pain and suffering. Of those, who have strength sufficient to arrive at manhood, the greater part are doomed to have little more than a glimpse of life, and to perish prematurely. Of those even, who appear strong and healthy, if we examine narrowly into their habits, or their feelings, we shall find hardly an individual, who will not acknowledge some defect, some secret uneasiness, something that diminishes his present comfort, and which excites apprehension for the future. In some, the solids destined to the support of the body are unequal to their object, and the bones yield to the incumbent weight: in others, the moving powers have a similar defect, the muscles hardly overcoming the resistance opposed to them. The senses are, in many, dull and imperfect; in many, they are preternaturally acute. The vital functions are often performed laboriously; the circulation is either sluggish or too rapid; the respiration straightened or hurried; the digestion is ill performed; the stomach oppressed with crudities; the secretions irregular; even the element, in which we are placed, appears ill suited to the organs, to which it is destined to be applied; some cannot bear the coldness of the atmosphere; to others its heat is equally intolerable; and so strangely constituted are individual constitutions, that an air loaded with mephitic vapours appears better suited to them, than one that is pure and uncontaminated.

"Man prides himself upon possessing an intellect superior to that of all other animals; and to take reason for the guide of all his actions. But, as far as happiness, or the mere absence of suffering, is the end of action, the reason of man appears to be inferior to the animal instinct. A brutal ignorance debases and enslaves the great mass of mankind. They appear incapable of acquiring knowledge; of perceiving the connexion of the ideas, which are laid before them; or the obvious relations of cause and effect. Thus they are void of all independence of thought or principle; a blind adherence to custom, or a slavish submission to authority, becomes the rule of life; and is substituted for self government, and a manly obedience to the voice of truth and the dictates of reason.

"The moral traits are as much distorted as the physical. The affections, which should link man to man, and make each human being regard his fellow creature as his brother, are choked and almost extinguished. Envy, hatred, jealousy, and all the malignant passions, predominate in the human bosom. The infliction of pain upon sensitive beings, instead of exciting compassion, is, with the multitude, a source of pastime and merriment. To such a degree are the strongest instincts of our nature perverted, that the first principle of self preservation is finally destroyed; the hand is raised against the existence of its possessor; or the parental arm against the life of the offspring.

"Such is an outline, too faithful, of the habitual condition, perhaps of the majority, of the human species. I omit the still darker darker shades of the picture; the tragedies which perpetually embitter domestic life; our crowded hospitals, from the gates of which, shoals of supplicants are, by necessity, repelled; our surgical operations, the very thoughts of which make the blood run cold; and our madhouses, the interior of which presents views, from which sensibility shrinks with horror and affright."

We shall not stop to ask the author, what proof he can find of any thing perfect in all that we know of mortality. These are not questions that can be solved, and, in these days, they rarely occupy the pages of philosophers or devotees. All we know is our own imperfection, and even in this we discover a first over-ruling and benevolent cause. We have said thus much, that we may not revert to the subject again, for it is perpetually occurring, and, where we least expected it, in so learned a writer.

The rest of the chapter is on the influence of air and water on the human frame, which, as Hippocrates was the first authority, is now traced in the writings of Linnæus, Lind, Trotter, Cabanis, M. Hoffman, Barton, Heberden, and Reeves; besides Bruce, Turner, Coxe, Staunton, Marsden, with other physiologists and topographical writers. From these the author concludes, that the sole cause of göitre is the water drunk in these places. The objections to this generally received doctrine are, it is true, stated; but, in our opinion, in a manner much more partial than we should expect from so honest, and, in other respects, so enlightened an author. We shall extract only a single sentence in explanation of our assertion.

"Others, (says Dr. L.) who speak slightingly of this opinion, content themselves with asserting, that the water was pellucid and well tasted. Such is the objection of Dr. Reeves; an objection certainly of very little weight, when unsupported by more particular examinations."

Now, Dr. Barton proves, that all these impurities of water, as far as they can be discovered, exist where göitres are rarely seen; and, it is well known, that the water about the Alps is particularly pure. But, on this occasion, the strongest argument produced by Dr. Reeves is entirely passed over, which is, that of the inhabitants drinking the same water, and breathing the same air; only those of a certain description, we might almost say, of certain families, escape; and that, in these, the females are more frequently affected than the males. If this would not lead us to impute disease to some family predisposition; an incident mentioned by the author himself, seems at least to encourage such an opinion.

"On this subject (says he,) I can speak a little from my own experience. In the parish of Horne, in the county of Surry, (a village six or seven miles to the south of Ryegate,) is the house of a laboring a laboring man, whose family consisted of five daughters; of these, four, whilst girls, became affected with bronchocele. In all, the disease was formed on this spot; but it continued, and even increased, after they had left it; going out to service. I saw one of them, a woman, perhaps of twenty-four, married in the neighbourhood: in her the gland continued swelled; but she said it was much diminished. The domestic water of this spot was a soft water, mingling readily with soap; it had a peculiar, and not agreeable taste; it deposited a small sediment by boiling, and showed (by oxalate of ammonia) a slight calcareous impregnation; but no more, probably, than is common to all the domestic waters of this country. I used the process described in another place, by which I determined that it was much impregnated with putrescent matter, which I believe to be much more noxious than the calcareous impregnation: and I doubt not, therefore, that this water had an active share in forming the diseased constitutions of these females."

Here then was a family complaint confined to the females. We have no account of any others affected in the same manner, though living in the same village, and drinking the same water. The girls too, were seized early in life, that is, before others exposed much longer to the imputed cause had felt such an effect; and, when these girls were removed from the imputed cause, the diseases continued and even increased. In the only one, as far as the account informs us, who married and remained on the spot, or in the neighbourhood, the gland is said to have been much diminished; in other words, the disease increased in those who, by engaging in service, were removed from the spot, and lessened in her who appears to have remained exposed to the imputed cause.

The fourth chapter is, in some measure, statistical. Mortality, we are told with much truth, is subject to fixed laws. This no true philosopher will dispute, but all will admit the great difficulty of detecting those laws, and the "number of erroneous opinions on that subject." The artificial nature and identity of constitutional diseases, is to us rather an intricate expression, which we have not found solved by our author, at least to our satisfaction. We meet, indeed, with many remarks so well established, that we are sometimes at a loss to know why they are produced. Let us take, for example, the following paragraph.

"It is obvious, from many considerations, that the quantity of mortality is quite inconsiderable, when compared to the general quantity of sickness; though this is a subject, on which it is impossible to form a calculation. Men are not always short-lived, because they are unhealthy: nor is great, and, apparently, very dangerous illness, in different stages of life, incompatible with arriving, finally, at old age. Little dependance, therefore, can be placed upon solitary observations with regard to the effect of particular ticular habits, or modes of treatment. Few are duly qualified to form a just estimate of such things. I am apt to think that, in this respect, even the sage Cornaro deceived himself. It becomes then of the first consequence to view mankind, as much as possible, in the mass, and to obtain, as far as it is in our power, general results."

On this passage we will offer a few remarks, which may save ourselves and our readers much trouble in the review of what may be not improperly called systematic writers. First, if the quantity of mortality is quite inconsiderable, compared with the quantity of sickness, we conclude, that most diseases cure themselves in spite of the doctor; and, as some new irritation will, for a time, usually supersede a chronic indisposition, we may thus account for the partiality each systematic physician has for his favorite remedy, whether purgatives, alterative doses of mercury, distilled water, James's powder, or any other preparation of antimony, tar water, Baume de vie, or, perhaps, a change from white to brown bread, or other apparently less important alteration in diet. Thus we perfectly agree with Dr. L., that little "dependance can be placed on solitary observations," and still more, that "few are duly qualified to form a just estimate of things;" that even "Cornaro deceived himself." Not, however, as we believe, in his own case, but in assimilating his own wants to those of the rest of mankind. It is not, therefore, to be wondered, if Dr. Lambe, like some others, should "unexpectedly meet Mr. Abernethy on a road where he did not expect to meet a surgeon."* "With regard to the generation of *constitutional* disease, (says Dr. L.) we may, I think, safely confine ourselves to four principal agents: these are, impure air, impure water, improper aliment, and fermented liquors." The last would have appeared to us a means of purifying water.

The author next considers the power of habit, showing that, in the animal part of man, nothing bad can become good or innocent by habit, though the immediate ill effects may not be perceived. Hence, that though men may accustom themselves to animal food, still that they suffer much by it. To the want of animal food, he imputes the mildness of the yellow fever, if it ever attack negroes. To the more sparing use of it, he imputes the greater exemption from the same disease that the inhabitants of the southern part of Europe enjoy, when transported to vertical regions; without considering that the change must be less in proportion as the subjects have been more accustomed to a warmer climate. Impure air, bad habits,

* Medico-Chirurgical Transactions, vol. vii. p. 513.

and and improper diet, in all great cities, we admit, may all contribute to the causes of greater mortality. But a regard to the profession obliges us to ask, what sort of company Dr. Lambe must have kept when he penned the following paragraph.

"One would be apt to imagine, from the common practice of most of our physicians, and still more of our medico-chirurgeons, that excess and intemperance were the regular methods of curing diseases. They have been laboring, during almost the whole of my medical life, to prove to the public, that the doctrines of abstemiousness, inculcated by several of our predecessors, are a mere prejudice and error. In almost all chronic diseases, to forbid the use of vegetables is a part of the established routine. If there be a little heart-burn or flatulence, all vegetables are instantly proscribed. Infants, even, are loaded with made dishes, and their breaths smell of wine and strong liquors. Nay, to such an excess are these abominations carried, that, when their stomachs revolt against these unnatural compounds, with instinctive horror, and the importunities of nature cannot be wholly resisted, a little fruit is held out to them as a sort of premium, and as a reward for forcing down the nauseous farrago, which they loath."

A number of instances are now recorded, in which temperance has produced very extraordinary effects in health and longevity. We are not in the least disposed to dispute any of them.

The objections made against vegetable food are next considered. "The paleness and loss of flesh." This is shown to be advantageous, and the well-known observation concerning *summa athletarum valetudo* is brought as an authority. The arguments, "that the feeble require nourishing diet; the differences of constitution; the uneasiness some feel from vegetables;" with many other carnivorous arguments or excuses are shown to be futile. All this might be well enough, but the conclusion of this chapter is really too bad for common stomachs in Great Britain. We are told, that all culinary vegetables are rendered less wholesome by boiling, and that it would be better we should eat our carrots and cabbages raw. This is, however, a little qualified afterwards, and the following conclusion of the chapter amounts nearly to an anathema.

"There may be other parts of our dietetic habits, which it would not be improper to examine. The use of tea and coffee, for example, is by many suspected, and, perhaps, not without reason. But I abstain from subjects, on which I am conscious that I have nothing of value to offer. I shall therefore conclude with making a single enquiry with regard to bread, which I shall leave to the determination of those, who are competent to pronounce on such questions, and who have proper opportunities of observations. What, I would ask, is this?—Is the farina of wheat, or any other, improved or injured, is it made more or less wholesome, by fermentation?

tation? or, in other words, which should be preferred, leavened or unleavened bread? The leavened or fermented bread sits lighter upon the stomach; but this is no proof that it is really more salubrious. We know very well that the coarsest black bread, which is as heavy almost as a lump of dough, gives much nourishment and strength. A sensible writer says, that he 'has heard a sea-faring man observe, that he was always sensible of a diminution of muscular strength, when he left off the use of biscuit, and eat common bread.'* A more venerable authority has given a corresponding testimony. His words are 'leavened or fermented bread is lighter in digestion, and passes easily through the body; but unfermented bread does not go off so easily, though it nourishes more, where the stomach can bear it.'†

"If these observations are correct, the fermenting of bread, and the cookery of vegetables, are practices adopted by mankind from the same motives: they accommodate the matters, to which they are applied, to the factitious delicacy of our digesting organs; which is effected, however, at some expense of their strengthening and nutritive powers."

The author improves much as he goes on. Though the effects of noxious habits may be slow, they are not less sure. Vegetable food is absolutely necessary to the perfect organization of man, and the want of it produces that imbecility so common in Iceland and Lapland. The natural progress of society is by an improved cultivation to render vegetable food more plentiful, and lessen the quantity of animals raised for food. In short, the use of animal food is a relic of barbarous manners. Of the readiness of barbarous nations to consume animal matter in any form, the reader will believe Dr. L. finds no difficulty in producing proof. We copy the following paragraph, leaving our readers to apply it to themselves, or the writer. It is well introduced by a quotation from that mystical work of *Plato de Anima Mundi.*

"It is allowed that men should be guided by reason: no truth can be more evident. But let us well understand what is meant by the term. By reason we cannot surely mean that feeble glimmering of light, which just enables the mass of mankind to grope through the gloomy paths of life, and to pass a few fretful years in a vain pursuit of happiness. The reason of individuals (if, indeed, it deserves the name) is commonly just sufficient to conduct them through the habitual occupations of the day: but the bulk of mankind are quite unable to comprehend the bearings of a complex argument; and still more to trace effects to their remote causes. Nor is this the case with the vulgar merely; for so limited is the

"* Dr. A. P. Buchan, in Sinclair's Code of Health, vol. ii. p. 109."

"† Hippocrates de Diætâ, lib. ii. x."

 human human capacity, that the most exalted genius, and the deepest powers of investigation, have not been able to raise their possessors above the errors and prejudices of their age, on subjects which have not been made the peculiar objects of their reflexions."

Our author now proceeds to some remarks on spirituous and fermented liquors, in which we feel no inclination to differ, thankful to find we are permitted the use of spices. If "man by nature is not a drinking animal," we conceive he must be out of his place in any but cool or humid countries.

Having thus gone through the general chain of reasoning, Dr. L. proceeds to offer several cases and observations in confirmation of his doctrine. These, however, are introduced by some very just remarks on the "errors and deep-rooted prejudices which pervade the general mass of mankind.

"Yet, hopes are still entertained, that, as the understandings of men become enlightened, beneficial changes may be introduced into the general habits of society. This is, however, a remote, and not a very cheering prospect. But to do all that is within the feeble powers of individual exertion to diffuse knowledge, and the blessings which follow in its train, is no more than striving to pay that immense debt, which every one owes to the community, who has received, from the sufferance of his fellow men, the exemption from servile and laborious occupations, and the inestimable advantage of mental cultivation."

The first case is of atonic gout. It is told with all the interest which the neatness of such an author's style never fails to produce; but, as it occupies thirty pages, we can only offer a few outlines. After suffering certain pains in the extremities, the head became affected, which induced the patient entirely to abandon animal food and fermented liquors. From this change no inconvenience ensued, but the relief was insufficient.

"In the month of May, 1803, he saw reason to believe, that deleterious matter was introduced into the body with the water that is habitually employed; and he determined, therefore, to try the effect of using none, but what was made perfectly pure by distillation. When he found that the uneasy state of the stomach was abated by this simple expedient, the delight received from the discovery may be more readily conceived than described. And, indeed, the real benefit produced was very considerable. He found a considerable improvement of muscular strength. In about nine months his sick headachs left him; and from that time to the present hour he has not experienced this great inconvenience once.* The constant uneasiness of the stomach soon become

"* He has been informed by others of sick headachs having been relieved by distilled water, particularly by a gentleman more than sixty years of age."

soothed, soothed, and in about fifteen months it was hardly sensible. All the dyspeptic symptoms were relieved, the stomach was no longer loaded and oppressed with flatulence; and the bowels performed their regular functions without the aid of medicine.

"Regularly in the month of October he had been subject, for some years, to severe attacks of pain in the jaws: so much that he used to take sixty, eighty, or even one hundred drops of tincture of opium to gain relief. This kind of attack recurred, the first year after the use of distilled water, with its accustomed violence. But since that time it has ceased entirely."

Still, however, disease occurred from year to year for nearly nine years after the regimen was begun. Without going through the whole, we shall only mark a few passages to show how "accidentally Dr. Lambe met a surgeon in the road," and to doubt whether a much more expeditious mode of cure might not be discovered by blue pill, or by purgatives, or some other favorite medicines, which would produce their effect in less than nine years, and without all this self-denial.

"Nor has the stomach (we are told) suffered from any vegetable matter, though unchanged by culinary art, or uncorrected by condiments. These results, so opposite to common experience, and even to his own, in the former part of life, can be accounted for only by considering the changes introduced into the state of the digesting organs by the previous use of the purified water."

That the blue pill is a much more expeditious and more agreeable remedy, we presume, not only because it effects in a few days what Dr. L.'s plan requires nine years to accomplish, and without these privations; but, because it does not induce those bodily sufferings in the periphery, which, notwithstanding all the privations, the patient is forced to undergo.

"To finish, therefore, this long account, (says our author):— After four years and a half, the gouty affection still continued, but its strength became so much diminished, that the lameness never again appeared. Sometimes there has been a slight stiffness of the heel; sometimes pains of the toes, with redness and soreness of them all. Through the whole of the seventh year (1812) there was a stiffness and some pain of the left knee. But, finally, in the eighth year, the whole of these external pains have disappeared, with the exception of that trifling affection of the head, which has been mentioned.

"Nor has this gouty disorder been the only external disease, which may be said to have been induced by the vegetable regimen. Formerly he hardly knew (as has been said) what it was to have a cough or a cold: the stomach or bowels were on all occasions of exposure the principal sufferers. But, at the end of the second year of the vegetable regimen, he had angina, infinitely more severe than he had ever suffered before. The attempt of swallowing was perfect agony. He has since had many severe coughs and colds, colds, attended with much defluxion. There has been also much itching on the surface of the body; particularly on the head, the hams, and the legs. But to compensate for these trifling evils, now the stomach and bowels never suffer."

By this passage, we maintain that the blue pill is by far the best remedy, as it not only restores the digestion, but prevents all other constitutional diseases, which induce local effect; and this, by Dr. L.'s confession, is much more than a nine years privation can promise. The next case is "Disposition to Phthisis Pulmonalis." The third pustulæ (pimples)." The fourth "Disposition to Apoplexia Hydrocephalica." Four cases of this kind occurred in one family, and were all successfully treated in a similar way. Another case of phthisis follows, after which we have the account of a brother author and reformer in diet, Mr. Newton, which is thus summed up.

"It is necessary, in order to form a fair judgment of this case, to pass in review its most striking points. They are shortly these: Mr. Newton began to use distilled water in 1805, and adopted the complete regimen in 1808. From this period of 1805 to June 1811, he had, upon the whole, very little asthma; hardly a single regular fit of any duration; and we were persuaded that the disease was in a manner eradicated. But, to our disappointment, and in a certain degree to our mortification, there has been, now for four years, an annual paroxysm declining, upon the whole, but not quite uniformly in severity. It has regularly come on in the month of June; which whole month it occupies, and encroaches a little upon July. Such is its present habit; and such we may suppose that for the present it will continue. I shall briefly attempt to explain these phenomena.

"First, it must be allowed, that the great freedom from asthma, for near six years, was not entirely due to his regimen. Diseases, we know, will change their forms. Asthma will end in consumption, hydrothorax, dropsy, disease of the heart, or other fatal maladies. It is obvious, from the delicacy of Mr. Newton's frame, and the great severity of his disease, that he is not formed, under common habits, for long life. I am, therefore, satisfied, that there was, about the time that Mr. Newton adopted a change of habit, some secret constitutional change, which concurred with his diet to keep off the asthmatic paroxysms."

As the author's theory appears to us quite new, we conceive it our duty to give it in his own words.

"I cannot (says he,) withhold offering in this place a conjecture with regard to the regular recurrence of the asthmatic paroxysm at the same period of the year, which has occurred now for four successive years.

"I suppose that it is allowed that the lungs themselves are the primary seat of the disease; and I will suppose further, that the membrane investing the bronchiæ and the air vesicles of the lungs is is the part immediately affected. It must be presumed that this membrane is liable to the same sort of diseases, as the other membranes of the body; but the consequences will depend upon the particular situation and functions of the part.

"Now, among other affections of membranes, there is one which, though very obvious, is not often adverted to; it is, that there takes place a species of exfoliation or sloughing; the membrane is destroyed, it is thrown off, and is regenerated. This whole process, of course, takes up some time; during which there must, of necessity, be a derangement of the functions, and a suffering of the individual.

"We see this phenomenon on the external surface of the body: the epidermis peels off; and, occasionally preserves its continuity, and the form of the part which it invested. It comes off the hand or foot, like a glove or stocking.* At other times it separates in flakes; which is a daily occurrence. But the intestinal evacuations give us more frequent and incontestible evidence of the same fact. Every one must have observed, occasionally, membranes evacuated preserving the form of the intestine. It is much more common, at the close of a diarrhœa, to observe a number of flakes, or films, floating in the liquid matter of the stool. This is commonly the solution and termination of the disease. These films can be nothing else than an exfoliation of the internal or mucous membrane of the intestine.

"It can hardly be doubted that the stomach itself is subject to a similar affection, though *it* is not possible to ascertain the fact by ocular proof. A person is seized with a constant vomiting, rejecting every thing which is taken into it, which lasts perhaps a month or six weeks. It will then cease, as it were spontaneously, and be no more heard of. What rational account can be given of such a phenomenon, unless it be what I have often suspected to be the fact; that the internal coat of the stomach exfoliates, and is regenerated?

"I have had reason to suspect that the bladder is occasionally subject to a similar affection: and, in general, that none of the mucous surfaces are exempt from it.

"We may readily transfer these observations to the mucous membrane lining the bronchiæ. It gives, I think, a more rational account of most of the phenomena of the asthmatic paroxysm, than any pretended spasm upon the vessels, or membranes. It accounts also, not inaptly, for the regular return of the disease. We know that the vital powers of all newly formed parts are weak. It is, therefore, easily conceivable, that under whatever circumstances the membrane has once perished and been regenerated, the same phenomena will recur under similar circumstances. It may be supposed to have received the same sort or quantity of vital power, as

---

"* Examples of this may be seen in Mangeti Bibliothec. Scriptor Med. tom. ii. p. 62.

as the horns of the stag, or the skin of the snake.—It is enough, however, to have thrown out the idea."

We are of the same opinion.

Another case of asthma convulsivum, with which Dr. Bree could do nothing for two years, was much relieved by this regimen in the course of ten months; and, after seven years' perseverance, the patient is only occasionally affected with pain in the side, and the bowels are not quite free.

We pass over a few other cases to make room for the chapter on Carcinoma, a subject which made a considerable part of Dr. L.'s former book,* but, in this, is confined to a chapter or two.

"I do not wish to conceal, (says Dr. L.) that the testimony which Mr. Abernethy gave to the accuracy of my statements, (as far as he was concerned) was given at my own request. For it is a fact, that Mr. Abernethy was so struck with the effect of the distilled water, in the case of cancer that he put into my hands, that he made upon it this pointed and remarkable declaration. 'I cannot be insensible, (he said,) to the effect of this treatment. Whether it will cure the disease or not, I cannot tell; but I can have no doubt that it will prevent it.'

"Mr. Abernethy, in consequence of what he saw, ordered the distilled water, at this time, in some other cases. One was a case of cancer of the rectum. It was a desperate case, in the very last stage of the disease; and the patient soon died. But the sufferer declared that it gave him much ease, and that it was the only thing, from which he had appeared to receive benefit. This declaration, or something tantamount to it, Mr. Abernethy told me, with the addition, 'that he should at all times be willing to acknowledge it.'

"This leads me to mention the circumstances, which induced me to be more sanguine with regard to the hoped-for result of cases, that were very far gone, than was justifiable by the event. I do this the more willingly, in order to guard others against a similar sort of deception, which will certainly occur again, under the same circumstances. What I allude to is as follows.

"In cases where the vital powers are greatly reduced, the evident change induced by a change of regimen, and the apparent advantage of such a change, is incalculably greater, than where the vital powers are more perfect; and where, consequently, the immediate danger of the patient is much less. This fact has appeared in a great variety of examples. I will cite a few that were remarkable.

"In a case of carcinoma of the mamma, a middle-aged woman adopted the regimen; and the consequence was, that the pain, which had been constant and severe for many months, was relieved, and almost removed in one fortnight. Such a circumstance could

* See our Journal, vol. xxi. p. 508.

not not but cause great delight, and excite hopes, that much good might be done in a short time. But these hopes proved fallacious. The woman died in less than six months; being cut off, as I judged from correspondence, by a peripneumonic affection.

"Another woman, laboring under ancites, received great, and almost instantaneous, benefit from the regimen. The abdomen began quickly to diminish in bulk, and for more than three months she appeared to improve in health daily. But then the benefit ceased, new symptoms supervened, and in less than another month she died.

"A little boy, of about four years of age, who was epileptic, was made to try the same plan of diet. The effect was highly pleasing, and even astonishing. After the course of a fortnight, the convulsions wholly ceased; and the head, over which he had appeared to have lost the power, became, in a great degree, upright. But he continued very stupid, with the sensibility so much impaired, that he seemed scarcely to be impressed even by fire applied to the skin. In about two or three months, the lower limbs became dropsical, the strength failed, and the child soon died.

"These, and several other similar events, have instructed us how little dependence is to be placed on the first changes, however imposing they may be; they soon showed that these sudden changes denote a great diminution of the powers of life, and would not have taken place, had the powers been perfect. In fact, the cases, which have ultimatively succeeded the best, have been those, in which the least benefit has been received suddenly: and from the repeated observation of such facts, I am now much better contented to be told, in a bad case, that little or no relief has been received, it may be, in several months, than the contrary.

"I have no doubt that the observation, which caused the acknowledgment, which Mr. Abernethy made to me, was similar to those which I have just mentioned. These declarations were made in the year 1805; and I was, therefore, not precipitate in expecting, that, when Mr. Abernethy was publishing on the subject of cancer in 1811, he should take the opportunity of acknowledging, that in the statement of facts, to which he had been a witness, I had been scrupulously observant of the truth. In that interval, the defect of the original proposal had been detected; and sufficient time had elapsed to have tried the power of the regimen; and to have ascertained, in a good measure, what it could really effect.

"But, though the recommendation which Mr. Abernethy gave was at my suggestion and request, he alone is answerable for the terms in which it was given. In particular, when he says, 'it is after an operation, that we are more particularly incited to regulate the constitution,' it is what I can by no means assent to. But more of this presently.

"Mr. Abernethy says also on this subject, 'I believe general experience sanctions the recommendation of a more vegetable, because less stimulating, diet, with the addition of so much milk, broth,

 an and eggs, as seem necessary to prevent any declension of the patient's strength.' On such a subject, Mr. Abernethy is, of course, much better informed than myself. But he certainly never informed me of this general experience; nor did I, during my attendance on the case, which Mr. Abernethy put into my hands, receive from him the slightest hint of such an opinion. No traces of such an opinion are to be found in Mr. Abernethy's works, previously published; not even in the second edition of his treatise; 'on the Constitutional Origin and Treatment of Local Diseases;' published when he had seen the progress of the case we attended.

"Nor was a diet of this kind recommended generally in cases of cancer even by Mr. Abernethy himself, previous to the publication of my 'Reports.' In proof of this I can say, the lady whom we attended, was eating animal food commonly twice a day; under the mistaken notion of supporting the strength, before it was resolved, at my suggestion, to change her diet, in February 1806. This was under Mr. Abernethy's own eye. I do not say it was done by his advice. He, I believe, never enquired into, nor gave any directions, on the subject. I will further say, that, had it not been for my strenuous application, this recommendation would not have been given, even in the place in which it has appeared."

Now, when we perceive this anxiety in claiming a discovery, we should really suppose that it was important proportionate to such anxiety; yet, reflecting that this important discovery concerning a painful and fatal disease, was published eight or nine years ago, and that the present work furnishes only a solitary instance of a scirrhous breast relieved; when, to this, we add the author's observation, that cancer, like all other diseases, is subject to infinite variety of forms; most of all, when we consider that this is the only instance we have heard of, except an authority we shall presently notice, we cannot help doubting the efficacy of distilled water in cancer. The authority we transcribe from Dr. L., with his remarks.

"'In scirrhous tumors, where the patients stamina is good, and particularly where the uterine secretion is regular, the vegetable diet and distilled water have proved very beneficial. The good effects of Dr. Lambe's treatment depend entirely on the natural stamina of the patient.'*

"I entirely coincide with this writer on this point. He has signed himself '*a Dispensary Surgeon.*' I am sorry that the author of this communication (which carries with it strong internal marks of correct observation) should have thought it proper to assume the mask of an anonymous signature; by which the weight which would have been attached to his evidence is considerably diminished."

---

"* The Monthly Compendium of Medicine, Surgery, &c. for December, 1809."

Can

Can there be wanted any authority to prove such a fact. Let the reader run over the passage again, and admire with us the readiness with which, and from what uncertain sources, the advocate for a remedy will receive the slightest intimation in its favor.

The other constitutional diseases in which Dr. L. has found advantage from his peculiar regimen, are cynanche laryngea, paralysis, local scrofula, rheumatism, prolapsus nasi, hypochondriasis, asthenia, physconia, leucorrhœa, calculus, carcinoma uteri, besides miscellaneous, communicated by different correspondents.

With much candour a chapter is added on the diseases which have occurred under the regimen. Some notes are attached to the end. The last relates to Mr. Hunter's doctrine and language, which are treated with much ingenuity: but we discover a very common error in confounding different *parts* of the *same order* with *different orders of parts*. We say nothing of the unfortunate expression from a scholar concerning the *eradication* of a disease, as if it had really taken *root*. These, however, are trifles, inasmuch as they are not necessarily connected with the object of the work; of which we trust we have given as fair and as perspicuous an account as our limits will permit.

---

*A Letter to the Right Hon. Lord Binning, M.P. &c. &c. &c. containing some Remarks on the State of Lunatic Asylums, and on the Number and Condition of the Insane Poor in Scotland.* By ANDREW HALLIDAY, M.D. Physician in Ordinary to his Royal Highness the Duke of Clarence, and Surgeon to His Majesty's Forces. Edinburgh, 1816. Pp. 34.

WE consider it an important part of our duty to notice books, the object of which is at once to alleviate human misery and improve the knowledge of medicine; and, when, as in the present instance, their contents are judiciously concentrated, so that the reader may readily peruse and comprehend the whole, we are relieved from the difficulties of offering any epitome of them, by producing such extracts as may show the manner in which they are executed, and the kind of information the reader may expect from them.

"From a review (says Dr. Halliday) of the number of insane in the country, and the accommodation of a public nature which at present exists, this active and benevolent magistrate [Sir William Rae] has sufficienfly proved, in his report to the Supreme Court of Justiciary, the impossibility of enforcing a proper attention to the comfort and cure of pauper lunatics in Scotland under existing circumstances.

circumstances. 'But,' says he, 'if certain public institutions existed, where lunatics could be received on moderate terms, the regulations as to private mad-houses might be made as strict as should be considered expedient, and their observance enforced with every degree of rigour. No complaint on the part of the keepers would then be listened to, as they might resign their occupation, and the public asylum would be ready to receive their patients. In England, such public institutions are established by two acts of parliament; the one 48 Geo. III. chap. 96. and the other 51 Geo. III. chap. 79. By these, each county is authorized to have such an asylum, the expence of the erection and maintenance being paid by the poor's rates; or two or more counties are allowed to join in erecting and maintaining such an institution at their joint expence. Such institutions, coupled with the proper inspection of private mad-houses, would certainly secure proper treatment to lunatics of all descriptions; but the one of these will not do without the other; and if the proper treatment of this most unfortunate description of persons is deemed worthy of attention, it is hoped, that the time is not far distant when these necessary means will be brought into effect. It would appear that there are not fewer than one thousand insane persons in confinement in this country, and that a very great proportion of these are actually supported by parish aid. For the proper treatment of such a portion of our fellow creatures, it is submitted that it much concerns the public that proper asylums should be found. It would only be requisite that the buildings should be erected at the expence of the district; for, with respect to the support of the institution itself, there seems no danger but that it would maintain itself. Patients, as to means, behoved to be of four descriptions: 1. Paupers supported by parish aid. 2. Such as have means of their own, or relations capable of paying nearly what is sufficient for their maintenance. 3. Such as can afford to pay more. 4. Such as cannot afford to pay so much.

" 'With respect to the first class, it ought to be made obligatory on all parishes within the district, to send their insane to the public asylum, and they should be bound to pay for them a sum precisely adequate to the expence of their maintenance. There seems no reason why they should pay less. In this way the maintenance of the 1st and 2d classes would cost nothing to the establishment, and the extra expence of the 4th would be more than defrayed by the surplus paid by the 3d class, and by donations, legacies, and other funds belonging to the institutions.

" 'Independent of the humanity of such a measure, there appears a clear principle for subjecting districts to the expence of providing such a place of confinement. In the case of other diseases, the public have no direct interest in attending to them, and they must consequently be left to the care of the humane and charitable. But in the case of insanity, the life of every man is put in peril; and accordingly, wherever a lunatic is found at large, the magistrate is called upon to interfere, so as to relieve the public from from the hazard they are thus exposed to. If the public are entitled to make this call, are not they bound to find the means by which such persons can be secured? A gaol is no way fitted for such a purpose. In general, the lunatic has committed no crime, and other prisoners ought not to be exposed to the annoyance or danger attending such an inmate. This leads to the observation, that there is at present no provision for the custody of criminal lunatics, the inconvenience of which is felt to a great degree. Such persons must either be left in jail, or entrusted to the uncertain care of friends; and the freedom which many of these enjoy, where this last mode is resorted to, doubtless has often had effect on deranged persons in the commission of crimes. There ought certainly to be one department in Scotland appropriated to criminal lunatics, to which they should be all sent, and from whence they ought never to be allowed to depart while in life. This might be connected with the asylum at Edinburgh; and government ought certainly to supply the means for its erection. In a letter from Mr. Clarke, treasurer to Bethlem Hospital, it appears that government gave to that hospital in 1807, 10,000l.; in 1811, 12,000l.; in 1812, 11,585l.; and in 1813, 39,234l. 1s. 6d.

"'Supposing the expediency of such institutions to be acknowledged, it may be right to look a little more closely to the means of constructing them. The objection to any measure whereof the expence must be defrayed by assessment is obviously that it falls heavy on the landed interest, and there certainly has seldom been a time when that interest has been less able to bear any additional burthen. But it does not appear to the Reporter, that the proposed burthen ought to fall on the landed proprietor. Insanity is an affliction which visits all ranks of society without distinction, and the expences of an establishment calculated for its relief, attach to all of these ranks. This principle was adopted in the Bridewell Act for the county of Edinburgh, being an establishment in like manner calculated for preserving the peace of all members of the community. This establishment is accordingly maintained by an assessment of one shilling annually on every house in the county of Edinburgh rented at above 5l., and a like shilling on every occupier of a plough-gate of land, form-houses not being charged; and this tax produces 650l. per annum. Supposing 5000l. wanted for the proportion for this county, it is obvious that two shillings on each house would produce 1300l. per annum; which, in four years, would more than give the sum wanted. If it was thought hard that all houses above 5l. of rent should pay the same, it might be enacted, that the lower class should only pay one shilling, while the higher paid three. But, in whatever way this might be arranged, the Reporter is confident, that, while such an assessment would completely accomplish the important object in view, its amount would be felt, or grudged, by no one. If this plan was gone into, it occurs that the management would fall to be vested in commissioners, who should have power to levy the rates, to apportion the districts, to approve of the plans, and to see them executed, to choose directors, and fix general regulations for their management, and to conclude agreements with private institutions now subsisting, so as to acquire right to the premises already erected by them, where suitable. It does not occur that there would be any difficulty in these arrangements, if the mode sanctioned by the English Act, where counties unite in the formation of such an establishment, there being one already subsisting in one of them, was adopted. Of such a commission, the Judges of the Court of Justiciary would naturally be the chief members; and provisions would be introduced, by which advantage might be taken of the presence of the judges at their circuits, correctly to ascertain the state of such asylums, and to see that the regulations were strictly adhered to. That court must have felt the difficulty which inferior magistrates every day experience, in having no place where criminal lunatics, or those wandering about uncared for by any one, can be committed. They must also be aware of the grievous effects upon any jail, of having lunatics therein imprisoned; and if, on the whole, it shall appear to that court, that either the remedy here suggested, or such better one as may occur to their judgment, ought to be adopted, it is hoped that they will avail themselves of the present moment, when the attention of Parliament is so particularly directed to this subject, to give such a recommendation as (when coming from such a quarter) cannot fail to be attended with effect.' "

"It appears, from authentic returns, that in eighty-five country parishes of Scotland there are not less than 387 individuals in a state of mental derangement;—that 102 of them are actually confined, either in private lunatic asylums, or, as is the case with by far the greater number, in the house of some friend or private person;—that only 31 are in public asylums, and that 4 are confined in county Bridewells or jails;—that 250 are permitted to wander about the country in a state of idiotism; and that 198, more than one-half of the whole, are maintained by the parishes or public."

From another return, it appears, that, on the 10th August, 1816, there are only *five* public asylums for the reception of lunatics in Scotland:—

| | |
|---|---|
| That the total number which these can accommodate is .... | 267 |
| To which may be added the Dundee Asylum, when completed | 40 |
| | 307 |
| That there are confined in these public Asylums, and in private Asylums, in the county of Edinburgh, with Bedlam and Charity Workhouses, not less than .................. | 412 |
| To which add the number from 85 parish returns actually confined, but not included in any of the places stated here .. | 102 |
| Total actually in confinement | 514 |

"I visited,"

"I visited," says Dr. Halliday, "the cells of the Edinburgh Bedlam a few days ago, accompanied by the celebrated German physician Spurzheim; and, although the appearance of the whole is much improved since I last saw them, yet it is impossible for language to depict their wretched state. We found *fifty-four* individuals in that abode of misery, two-thirds of them females; many had scarcely a sufficiency of rags to cover their nakedness, and even the shreds that remained appeared not to have been cleansed of their impurities for months. In a distant cell we discovered a woman, worn out by the violence of the disease, stretched upon a straw pallet, and sinking rapidly to the grave. A *rat* was perched upon her bed: I shall not affirm that this animal had attempted to mangle the exhausted body of the dying maniac; but the sight was horrible! Alarmed by our unexpected intrusion, it retreated coolly through a *large hole* in the floor of the cell."

A short paragraph follows, which concludes the work with the following pathetic sentence:—"I am grieved," says the author, "to add, *the swine in Germany are better cared for.*"

---

### *Edinburgh Medical and Surgical Journal*, No. L. April, 1817.

Art. I.—*Critical Review of the State of Medicine during the last Ten Years* (article concluded).

In our remarks on the last number of this work, we made a sort of promise to give an abstract of the "Critical Review of the State of Medicine during the last Ten Years," as it was to be completed in the present number. We have no scruple to say, that our readers will readily relieve us from such an engagement. Notwithstanding the frequent exercise of our patience, this is really too much for us. Let, then, this notice serve as a record, where such a review of German lumber is to be found. The learned Editors have prudently already so much shortened it, that little more than an enumeration of authors and their works can be collected from it.

Art. II.—*Reports on the Ardent Fever of the West Indies, as occurring on board His Majesty's Ships Raven and Niobe, in the year* 1815. By Mr. Peter Comrie, Surgeon, Royal Navy.

This account is truly valuable; and we could gladly go over the whole, were it not that the author's premises have been already so well established. We may, therefore, content ourselves with a few remarks. The number of cases which came under Mr. Comrie's care was 172; the number of perfect recoveries 166. In all the successful cases, early bleeding, bleeding, not regulated by quantity but by effect, was the remedy. In some cases more than 200 oz. in two or three days, besides other evacuations and cold affusions; or, if the rigors continued long, the warm bath. In the case which terminated fatally, these remedies could not be applied early enough. As they are few, and told with brevity, we shall subjoin notes of the whole, six in number.

"Case I.—John Herrop, ætatis 34, of rather a spare habit and dark complexion; had a severe attack of fever formerly. Was ill two days without complaining. First seen on 11th December. Evacuations were freely used after he applied, but without effect, and he sunk on the 13th at midnight.

"This man's case was remarkable as being unaccompanied with the delirium, dark-coloured vomiting, and yellowness of surface, which were constant symptoms in all the other fatal cases.

"Case II.—Robert Williams, ætatis 20, of a sallow complexion, stout made, and somewhat plethoric; had been severely attacked with the first endemic that appeared in the Niobe, and sent to the Royal Naval Hospital at Antigua. This time he was indisposed for some time previously without making application, until the 14th December, in the evening, when I found him affected with the usual symptoms of pyrexia. In this case, after application, the antiphlogistic regimen was most strictly used; but all efforts were unavailing, and he expired on the 19th December, at 7h. 50m. P. M.

"Case III.—John Jones, ætatis 28, very stout made, about six feet in stature and stout in proportion, of a dark complexion, and somewhat plethoric; was indisposed one whole day, without making application; but had not been previously sick since I joined the ship. Complained, on the 15th December, of the usual symptoms of fever. With very great persuasion, I prevailed upon him to allow me to detract only about 32 ounces of blood, and he would not afterwards consent to let any more be taken away. At that time I was very much occupied, and had not leisure to use much persuasion in order to induce him to submit, as I had others to attend to, who allowed themselves to be treated as I thought proper. He expired on the 19th December, at midnight. Some days before death he vomited a vast quantity of blood.

"Case IV.—Joseph Hurst, ætatis 28, very much emaciated, and addicted to intemperance. Was severely attacked with the first endemic that appeared in the Niobe, and sent to the Royal Naval Hospital at Antigua, where his life was for some time despaired of. Was attacked on the 15th December with fever, accompanied with delirium. I only detracted about 20 ounces of blood at the commencement, and was afraid to take any more away, dreading he would sink under the operation, and the remedy be disgraced, and thereby others be deterred from submitting to it. He expired on the 19th December, at 9h. 30m. P. M.

"Case V.—John Taylor, ætatis 23, stout, red-haired, and plethoric; plethoric; was attacked, on the 15th December, with the usual symptoms of pyrexia. He was attended by the assistant-surgeon of the 1st West-India regiment; but evacuations were not so freely used as I directed, and through fatigue and indisposition I was not able to attend him and some others as I would have wished. He died on the 23d December, at 9h. 30m. P. M. dreadfully delirious.

"CASE VI.—Bartholomew Rodgers, ætatis 44, stout made, red-haired, and somewhat plethoric, and had been a long time previously in the West Indies. He was attacked with the first endemic that appeared in the Niobe, and sent to the Royal Naval Hospital at Antigua, where his life was sometimes despaired of. Had been indisposed for two or three days before I was informed thereof. December 16, when I first saw him, he was confined to his hammock in the lower deck, with symptoms of a severe fever. Though evacuations were pretty freely used, he expired on the 18th December, at 7h. 40m. A. M."

We presume the severe duty of the author, and, probably, his own want of health, prevented his examining any of the bodies. At the same time we must admit that the appearances are now pretty well ascertained.

By the above it appears that four of these subjects had been attacked during a previous epidemic, and perfectly recovered, as the table shows. It is not for us to say how far this proves that the yellow-fever may affect the same person twice, nor shall we undertake to determine whether this disease, called by Mr. Comrie, with much caution, "ardent fever," was really the yellow-fever; nor whether there is sufficient difference in the type of fevers of the West Indies to be thus distinguished, whatever may be the difference of cause. The pain was the principal symptom which indicated bleeding.

"When (says the author) the disease terminated favourably, particularly in those cases where the abdominal viscera were affected, I have seen, after venesection, the pulse become most remarkably full and frequent, but very soft; at first generally about 120 or more, then about 96, and afterwards gradually regular.

"This state of the pulse, when it was observed, came on about 12 or 24 hours after all bleeding was laid aside, and was always a favourable symptom; but, when I felt it first, I was nearly deceived by it, and was going to abstract more blood, which I think would have done much harm. I desisted on the patient mentioning that he had no pain, and upon perceiving that he had rested well, and had a pleasant countenance."

The last remark we shall make is the success with which the invasion of fever was checked by the resolution of the Captain to steer northward on its first appearance. Though less less than twenty degrees from the equator, we shall see the happiest effects followed.

"The ship's company, since I joined this ship, on the 28th May, 1815, has been, comparatively speaking, pretty healthy, until we arrived, on the 14th September, at English harbour, Antigua, to refit, where we remained until the 25th. During this interval a great many of the ship's company were attacked with the endemic of this country; and, Captain Deacon seeing it increase more and more, and being informed, that, as it increased, it was attended with more violent symptoms, with much prudence and discernment went to sea, and cruised to windward of all the islands, and until we were in latitude 19° 39′ north, and longitude 61° 45′ west, when the fever began to decrease. We then returned to English harbour to complete the refitting of the ship, and scarcely any were again affected with fever, although we remained at English harbour from the 3d of October to the 14th, and although the ship's company, watch by watch, had liberty to go on shore frequently."

On the whole, we consider this a very useful paper, and trust it will have its proper effect in establishing the only reasonable plan of treating tropical fevers in new comers.

Art. III.—*Case of Enlargement of the Heart with Polypi, &c. which occurred on board the Hon. East India Company's Ship Carnatic.* By James Stewart, Surgeon.

As we mean to make some observations on this case, we shall transcribe it at full length.

"May 5th, 1816, William Cooper, seaman, aged 30, middle stature, peculiarly full broad chest, fair complexion, and plethoric habit of body; since leaving Bengal, about three months ago, has not enjoyed his usual state of health; appetite being variable, and a dull pain, or rather sensation of weight under the ribs of his left side, frequently annoying him. These complaints were, however, so trifling, as not to prevent him from doing his usual duty. A few days ago, he was very suddenly attacked with a severe pain in the left hypochondrium, which was nearly removed by the operation of a smart purgative, followed by a diaphoretic. This morning it has again returned with increased severity, and now extends to the front of the left shoulder; not increased on pressure; pulse not accelerated, but full and strong; tongue furred brown; considerable thirst; countenance pale, and features shrunk.

"Capt. calomel gr. viii. pulv. jalap. ʒſs. misce et applicat. emplas. lyttæ part. dolent.

"May 6th. After the operation of the purgative and blister, the state of his tongue improved considerably, and the pain almost ceased.

"Last night again, a severe exacerbation suddenly took place, and and nearly as suddenly ceased, about seven hours afterwards. During the paroxysm, a draught of tinct. opii and æther sulphur. was given him without any beneficial result. The situation of the pain had, however, now changed to the region of the heart; no pyrexia; general health little affected; pulse to-day 88, full and sudden, but perfectly regular.—Detrahat. sanguis ad ℥xxiv.

"May 7th. Nearly free from pain all yesterday. During the night a smart exacerbation again occurred, and situated immediately over the region of the heart; sympathetic affection of his shoulder annoyed him much; cannot now lie on his left side, and feels most easy when leaning forwards, or lying on his breast with his arms extended, so as to enlarge the cavity of the thorax. During the attack of last night, his countenance was pale, and features shrunk, with increase of thirst, and foulness of tongue, but scarcely any change in the temperature of his skin, or state of his pulse.

"Habt. stat. ext. colocynth. comp. et calomel āā gr. vii. et repet. V. S. ad ℥xvi.

"May 11th. Scarcely any pain in his arm since last report, but his shoulder has been extremely uneasy with a sensation of numbness, extending nearly to his fingers; he has been using stimulating anodyne liniment, without relief; tongue more foul; spirits unusually depressed; pulse 80, soft and regular; gave him yesterday seven grains of pilul. hydrarg. which procured three easy evacuations. Repet. pilul. hydrarg. et cont. liniment. anodyn.

"May 12th. Yesterday evening, had a slight increase of pain in his breast, with a most tormenting uneasiness in his shoulder and arm. Pulse was 86, more soft and natural than usual. Countenance and spirits were much depressed; foulness of tongue and thirst increased. Gave him gr. vi. calomel, and gr. v. pulv. scammon. which soon excited vomiting, about an hour after which, the medicine beginning to operate, he went upon deck, where he dropped down and expired without a struggle.

"*Sectio Cadaveris.*—Upon opening the thorax, observed the pericardium prominent, and occupying a much larger space than natural; its cavity was found to contain about fifteen ounces of serum, with two ounces of coagulated venous blood, discharged from some of the small vessels on the surface of the heart, all of which were peculiarly enlarged and turgid. The heart itself was preternaturally large, weighing at least 2½ pounds. A small surface over the left ventricle showed marks of slight superficial inflammation having existed, and around the exit of the aorta from the heart, an effusion of blood into the cellular substance, to the extent of about half of an inch, had taken place. On examining the interior of the heart, the size of the cavities, columnæ carneæ, &c. corresponded with its external magnitude. The right auricle, right and left ventricles, contained each a large polypus, attached by three or four pedicles. The one in the right auricle weighed upwards of an ounce, the others about an ounce each. They were of a pale red colour, a solid firm consistence, and, when cut into, presented a highly vascular and organized appearance, so that no doubt remained, or yet remains, on my mind, as well as the mind of another medical gentlemen present, *that they must have existed a considerable time previous to death.* The one in the auricle was lying against the mouths of the cavæ, and consequently opposed to the course of circulation. That in the right ventricle, of an oblong shape, was carried by the current of blood into the pulmonary artery; the other was firmly bound down in the left ventricle by its pedicles. No other morbid appearance presented itself in the thorax. In the contents of the abdomen, nothing peculiar was observed.

"This case I have wished to make known to the public as presenting some symptoms which I conceive worthy of particular notice; and also as a case of polypi, which, from their vascularity and perfect organization, must have existed for a considerable time previous to death. From the seat of the pain in the left hypochondrium, when he first applied for assistance, I conceived his complaints to arise from some visceral obstruction, the consequence of service in tropical climates. Its removal shortly afterwards to the region of the heart, and especially the occurrence of the sympathetic affection of the left shoulder and arm, soon excited suspicion that the *heart* was the seat of his disease; still, however, the pulse remaining at all times perfectly regular, and being seldom if ever accelerated, led to some obscurity with respect to the nature of his complaints, and doubt how to proceed in the treatment."

How much we could wish that this gentleman had, like the author of the preceding paper, judged by the pain instead of the pulse. We must commend him much for his examination of the body, and question not his faithful detail of appearances. But, after a careful perusal, we can find no reason to suppose that these polypi had been of long existence. Inflammation, which would produce the effusion of lymph so firm and so free from red particles as be of a *pale* red colour, would *instantly* organize such effusion by the formation of vessels. The author, if we understand him, seems to conceive that these vessels are mere elongations of those from the surrounding parts, to which the polypi adhere. But this is by no means certain, nor, if it were, is it any proof that the coagula had existed long. Indeed, if the process of thus organizing polypi was slow, they must lose their life, and become sloughs. How often have we to lament the little attention paid to the pathological doctrines of Mr. Hunter. Our 20th vol. p. 538, contains the description of a somewhat similar appearance. We shall extract a single paragraph, but recommend the perusal of the case to those who have any doubts that such vessels may be formed with with the rapidity for which we contend. The subject died after five days' illness, during which the pain was very violent in the region of the heart.

"The right auricle contained a large yellow coagulum, almost filling the cavity adhering to it, and containing blood-vessels which communicated with the substance of the auricle. This coagulum was continued to the ventricle in an oblong form. To this cavity it was more firmly attached by communicating vessels. There was also a considerable membrane of effused lymph adhering to the inner surface of the right ventricle."

In this case, the coagula were so large as very much to impede the action of the heart and the passage of the blood; so that we should rather wonder the patient lived so long, than that he died so soon and so miserably. The most sceptical, we conceive, will not suspect that, in this case, the polypus *had existed a considerable time* previous to death. On the subject of the early formation of blood-vessels in coagula, see Mr. Hunter's Treatise on the Blood, p. 92, 4to. edition.

Art. IV.—*Case of Pneumonia, with Observations on the Practice of Blood-letting in that Disease.* By Wm. Gibney, M.D. Assistant-Surgeon 15th King's Hussars.

Art. V.—*Reflections on the Arrangement of Cutaneous Diseases; with the suggestion of a Practical and Diagnostic Mode of Classification.* By Marshall Hall, M.D. &c.

More hard names! We have often expressed our conviction that our knowledge on these subjects is much too imperfect to attempt any artificial arrangement. Dr. Bateman admits as much. The itch and syphilis, the two most common of them all, he observes, bid defiance to such attempts: Why, then, do we undertake it in others? Is it not certain that the same cutaneous disease appears differently on different days, or different parts of the same day? There is, however, one advantage in Dr. Hall's arrangement,—that he separates acute from chronic complaints. This is the more necessary, because on the former we must be decided, and must examine the appearance from day to day. In the latter we may take more time, and the appearances will vary less.

Art. VI.—*Remarks on the Preservation of Lime-Water.* By H. Dewar, M.D. &c.

These remarks are simple, and easily reduced to practice.

"Wishing (says Dr. Dewar) to have a quantity of good lime-water constantly at hand, for the sake of experiments, I have found found the following method completely satisfactory:—Instead of filtering the solution, to keep it in contact with a quantity of quick-lime, in a tall wide-mouthed bottle, and to decant from the upper part, as much as is at any time wanted; to fill the bottle immediately again with water, and shake it in the same manner as when the lime-water is first made. This excludes all carbonic acid, and affords a fresh supply of the solution. It is preferable to the plan of keeping quick-lime bottled up in a dry state, which is apt to break the bottles, and, when at any time exposed, exhibits a more extensive surface to the action of carbonic acid.

"When the simple plan now described is followed, even though precipitation by the carbonic acid should, in a slight degree, take place, the solution, thus weakened, is quickly re-saturated by the presence of the quick-lime. Thus it is always kept in its strongest state, and may be obtained perfectly limpid. The trouble of inquiring frequently for fresh burned limestone, which, in some situations, may be considerable, is avoided, as well as that of filtering. This would, indeed, be a paltry recommendation, were it not that the trouble is of no utility, every object being more advantageously obtained without it. The filtering, besides, implies a degree of exposure which must be injurious.

"I can conceive no objection to which this little plan of chemical and pharmaceutical economy is liable. On the contrary, I trust that it requires only to be mentioned to have its advantages appreciated, and to be generally adopted. Apothecaries and druggists may keep their stock in very tall and wide-mouthed bottles, with quick-lime at the bottom, from which it may from time to time be drawn off, either by the use of a syphon, or by careful decantation, and put aside in vials containing the quantities most generally asked for."

Art. VII.—*An History, with Remarks, of an Aortal Aneurism, conjoined with a Pulmonary Phthisis.* Extracted from a Clinical Lecture, delivered February 10, 1817, by Geo. Pearson, M.D. F.R.S. &c. in a Letter to Andrew Duncan, sen. M.D. Professor of Physic, &c. &c.

The reader will perceive something peculiar even in the title and mode of communication of this paper. The paper itself abounds, if we may so call them, with oddities. We shall first give the outlines of the case which is certainly not without its interest, and then point out what appear to us the peculiarities of the manner in which it is announced and related.

The patient, a foreigner, had, by his own account, felt no illness till September 1816, yet died of ruptured aneurism of the aorta on the 31st of January following, a period at most of not more than five months.

"On admission, the patient seemed to be in a dying condition, so that no distinct disease could be ascertained. The dyspnœa was considerable, considerable, but the weakness seemed to be so great that he was unable to expectorate, and only made efforts to cough. The beating of the heart and pulse at the wrist were very feeble, but synchronous.

" Wine, cordial medicines, and nutritious matter were ordered, merely to palliate and support life.

" Contrary to my expectation, strength was gradually recovered. The breathing became easy while recumbent on the back; could also lie on either side, but not comfortably; became able to cough and spit up much muco-purulent matter. The pulse was between eighty and ninety in each minute, but sometimes not more than seventy-five; the tongue from foul became clean; little complaint was made of pain of the region of the heart, but there was a feeling of obstruction in its pulsation. The countenance looked materially better in four days' time. However, on the 30th December, there was much pain of the left side, and a good deal of difficulty in breathing, with frequent coughing. The wine and cordial medicines were discontinued.

" Blood-letting to ten ounces was ordered; a purging draught; a blister to the left side; and an opiate at night.

" The pain of the left side continuing, although the dyspnœa was relieved, the blood-letting was repeated; but the blood was not buffy.

" In addition to the pleuritic pain, much complaint was made of pain between the shoulders. The painful feelings subsequently to about the 7th January, were confined to the region of the heart, which increased, the hand being frequently applied to it to denote the seat. More and less sputum continued to be coughed up; weakness increased; blistering plasters were repeatedly applied, and, from the urgency of the pain, also blood-letting; but the blood was never sizy. Ipecacuanha, squill, opium, and demulcent medicines, were the internal medicines recommended.

" From the 20th January all the symptoms grew more and more unfavorable; then much distress was denoted by the countenance; great restlessness; the body was often desired to be raised, and also bent forward from time to time to relieve the anxiety and painful feelings of the chest, but the weakness confined the patient generally to lying upon the back; the respiration grew quick; the cough more troublesome; the sputum as before; the appetite for food totally failed, and the thirst, with foul tongue, increased.

" One night, in a fit of delirium, the sufferer suddenly got out of bed, and walked about the room, saying he 'was now well.' It was particularly observed, that the pulse varied only between eighty and ninety, excepting rarely under this rate. It had been also observed, that no morbid action took place of the heart, excepting a little throbbing.

" On the 31st January, in the evening, during a slight coughing fit, a little blood was expectorated with the muco-purulent matter; and the patient expired almost without a struggle."

What was this muco-purulent matter? Is it the same as the the sputum? and, if so, why too not very common expressions?

"*Dissection.*—About sixteen hours after death the chest was opened. The lungs appeared of a whitish color, like those of brute animals which die by hæmorrhage, as commonly seen in butchers' shops. There was less charcoal than common on the exterior parts, but the bronchial glands were black as usual. The left lung was contracted to nearly half its natural bulk; it felt firm instead of spongy, and totally sunk in water. The right lung filled the cavity of the thorax; was extremely light; contained much air, which readily passed from one part to another by slight pressure. To the extent of several square inches blood was effused under the pulmonary pleura from a blood-vessel, probably ruptured on the surface. Though double the bulk of the left lung, it weighed rather less, namely, fourteen ounces. There were no adhesions nor membranes thickened in the thoracic cavities; but there was about half a pint of serum in the left cavity: in the right was most unexpectedly found a large quantity of black blood slightly coagulated, amounting to about two pounds, besides half a pound of serum. This blood, after standing exposed to the air twelve hours, exhibited a thin stratum of red blood on its surface, as in the case of venous blood so exposed and coagulated."

Here again, "animals that die by hæmorrhage" why not "are bled to death?" Some of our country readers will also ask, what this expression of "less charcoal on the exterior parts" means? and how much charcoal we ought in general to expect. The blood seems to have required two hours to acquire that redness on its surface, or, as the writer says, "to exhibit a thin stratum of red blood on the surface." Now the surface of blood, whether arterial or venal, (for arterial blood becomes black by exclusion from air,) in general becomes red a few minutes after such exposure.

The heart and right lungs were in a natural state.

"The left lung was also found almost bloodless. The air tubes were filled with pus throughout, and innumerable tubercles, of the size of pepper corns, were found in the substance of this viscus; but there were no vomicæ nor large masses of coalesced tubercles, nor masses of condensed lung without tubercles."

"The source of the effused venous blood was the next object of inquiry. It was discovered to be from a large aneurismal cyst of the aorta, just below the descending *arcade*. This cyst, several inches in width and breadth, had been burst in its superior part; and so had the contiguous membranes of the posterior mediastinum into the right cavity of the thorax; and of course the gush from an aperture nearly two inches wide, instantly drained the lungs and heart of their blood; probably, too, blood was transmitted through the lungs for a very short time after respiration had ceased, and hence the great quantity of effused venous blood. In the ruptured sac was contained a firm reddish coagulum of a roundish figure, which, being easily detached, was found to weigh eight ounces. It consisted of laminæ or leaves, which, with little trouble, were separable of such tenuity as to be semitransparent. By maceration in cold water they became white. Depositions of the self-coagulable, or live lymph of the blood, had probably successively taken place from the bottom of the cyst, in an horizontal position, till the large mass of layers occupied great part of the cavity."

That the coagulum in an aneurism should be in layers, is not surprising, nor that these layers should become white with washing. But, why these expressions of *self coagulable* or *live lymph?* If the adjectives ending in able are passive, as in *portable*, *potable*, &c. we may as well talk of a burthen which may be carried by itself, a liquor which may be drunk by itself, as blood which may be self coagulable. If the lymph of the blood is alive, why should we not use an active participle? But the writer might have saved himself all this trouble and intricacy, by describing the lymph simply as *coagulated*. This would be enough to explain its appearance, and if he wished to do more, he should have gone much further into the subject concerning the formation of these coagula. With this view, we would recommend to this pupil, we presume, of Dr. Pearson's, that he should peruse the late Dr. William Hunter's paper on Aneurism, in the first volume of "Medical Observations and Inquiries." He will then see, if not an explanation, at least an anticipation, of what seems to puzzle and surprise him so much.

"A most striking effect of the pressure of this aneurism was the entire disappearance of the osseous matter of the anterior part of the second, third, and fourth dorsal vertebræ, although the cartilages remained whole: thus leaving large cavities from the loss of bone between these cartilages by absorption. The absorption of bone, but not of cartilage, although subject to equally great pressure, was noticed as a curious pathological fact."

Lest our youth should not have the book at hand, we will transcribe the passage alluded to.

"Of all animal substances (says the author of the paper in the Medical Communications and Inquiries, vol. i. p. 348,) gristle, perhaps, is the least affected by pressure in the living body. This seems probable from the known structure and uses of the joints, but was particularly evinced by the above case. The constant pressure of the sternum had destroyed the coats of the artery, the periosteum, the bones, &c. except the cartilaginous part of the ribs. These were pushed to one side indeed, but almost perfectly sound in their texture."

We could make several other observations on the rest of the the paper, but trust, what we have said, will be enough to induce this student in future, either to give papers of his own, or to transcribe his master's opinions with more accuracy. We would further recommend him to lose no opportunity of examining bodies, the previous history of which he may be acquainted with. He will then feel less surprised, that a very experienced and accurate physician, one of the first anatomists in London, should treat, without any suspicion, a case which proved to be aneurism. Nothing but the inexperience of youth could excite a wonder on these subjects. We cannot conclude, without expressing our satisfaction at this opportunity of offering wholesome advice where we trust it will not be entirely lost. There are evident marks of genius in the paper; and we should be sorry to discourage the writer from offering future communications.

### MEDICAL AND PHILOSOPHICAL INTELLIGENCE.

*To the Editors of the London Medical and Physical Journal.*

GENTLEMEN,

